# First Molecular Detection and Characterization of *Nosema ceranae* in Honey Bees (*Apis mellifera*) from the Northern Highlands of Ecuador

**DOI:** 10.3390/insects17030302

**Published:** 2026-03-11

**Authors:** Dayana Sandoval-Morejón, Cristina Cholota-Iza, Marbel Torres-Arias, Karina Antúnez, Armando Reyna-Bello, Luis Fuentes-Hidalgo, Claude Saegerman, Sarah Martin-Solano, Jorge Ron-Román

**Affiliations:** 1 Laboratorio de Biotecnología Animal, Grupo de Investigación en Sanidad Animal y Humana (GISAH), Departamento de Ciencias de la Vida y de la Agricultura, Universidad de las Fuerzas Armadas ESPE, Sangolquí 171103, Ecuador; edsandovalm@uce.edu.ec (D.S.-M.); cecholota@espe.edu.ec (C.C.-I.); aareyna@espe.edu.ec (A.R.-B.); ssmartin@espe.edu.ec (S.M.-S.); 2 Centro de Biología, Universidad Central del Ecuador, Quito 170515, Ecuador; 3 Laboratorio de Inmunología y Virología, Grupo de Investigación en Sanidad Animal y Humana (GISAH), Departamento de Ciencias de la Vida y de la Agricultura, Universidad de las Fuerzas Armadas ESPE, Sangolquí 171103, Ecuador; mmtorres@espe.edu.ec; 4 Centro de Nanociencia y Nanotecnología, Universidad de las Fuerzas Armadas ESPE, Sangolquí 171103, Ecuador; 5 Departamento de Microbiología, Instituto de Investigaciones Biológicas Clemente Estable, Montevideo 11600, Uruguay; kantunez03@gmail.com; 6 Laboratorio de Mejoramiento Genético y Sanidad Animal, Grupo de Investigación en Sanidad Animal y Humana (GISAH), Departamento de Ciencias de la Vida y de la Agricultura, Universidad de las Fuerzas Armadas ESPE, Sangolquí 171103, Ecuador; luis27fuentes@gmail.com; 7 Research Unit of Epidemiology and Risk Analysis Applied to Veterinary Sciences (UREAR-ULiège), Fundamental and Applied Research for Animal and Health (FARAH) Center, Department of Infections and Parasitic Diseases, Faculty of Veterinary Medicine, University of Liège, 4000 Liège, Belgium; claude.saegerman@uliege.be

**Keywords:** *Microspodiria*, nosemosis, *Nosema ceranae*, *Nosema apis*, molecular diagnosis, epidemiological investigation, fluorescence microscopy, *RPB1* gen, *16S rRNA* gen

## Abstract

The development of beekeeping in Ecuador has generated the need to strengthen the bee health program. Research on the main pathogens responsible for diseases like nosemosis, which can severely impact bee health, is of special interest. This study aims to identify the *Nosema apis* and/or *Nosema ceranae* species infecting honey bee colonies located in the northern Andean region of Ecuador using multiplex PCR targeting the RNA polymerase II gene (RPB1), and the phylogenetic analysis of *N. ceranae* based on the 16 S rRNA gene sequences. Among the 164 honey bee samples collected from colonies in the provinces of Carchi, Imbabura, and Pichincha, the prevalence of *Nosema apis* and *Nosema ceranae* was 14.63% and 21.34%, respectively. Phylogenetic analysis showed that *N. ceranae* from Ecuador is closely related to the sequences from Argentina and Brazil. These findings provide the first molecular confirmation of *N. ceranae* in Ecuador and support the need for molecular monitoring of honey bee pathogens in the region.

## Simple Summary

Bees play a key role in agriculture and the environment since they pollinate many plants that provide food for people and animals. However, their health can be affected by microscopic parasites that cause diseases and weaken colonies. In Ecuador, little is known about which of these parasites are affecting honey bees. This study investigated the presence of two species of *Nosema*, a group of tiny organisms that infect bees and can reduce honey production and colony survival. Samples were collected from different provinces in the northern region of the country, and laboratory tests showed that both species, *Nosema apis* and *Nosema ceranae*, are present in Ecuador. The second species was found more frequently and is closely related to those found in other South American countries. This is the first report confirming the presence of both *Nosema* species in Ecuador. These findings have implications for food security and environmental sustainability.

## 1. Introduction

Beekeeping activity in Ecuador has been growing steadily. In 2016, a total of 902 apiaries and 12,188 colonies of domestic were registered, with most of them concentrated in the Sierra region (mountain area), where the provinces of Pichincha (22.79%), Imbabura (8.41%), and Carchi (7.99%) have the highest numbers of colonies [1]. Given the increase in this activity in the country, the Agencia de Regulación y Control Fito y Zoosanitario de Ecuador (AGROCALIDAD) aimed to obtain information regarding the health status of those colonies. They conducted a nationwide study of the main pathogens affecting honey bee colonies and reported the presence of *Nosema* sp. in 235 apiaries [1]. However, a molecular species differentiation is lacking.

Microsporidia of the genus *Nosema* are obligate intracellular parasites [2] comprising more than 150 described species [3] affecting both mammals [4] and insects [5], particularly those of the orders Hymenoptera and Lepidoptera [6]. Nosemosis is a disease caused by the microsporidia *Nosema apis* [7] and/or *Nosema ceranae* [8,9,10] with a worldwide distribution [11,12,13,14,15], and is recognized as an important contributor to colony weakening across diverse geographic regions. Although both species have recently been reclassified under *Vairomorpha* [16], this reclassification remains under debate [17]. Therefore, both the traditional designation of *Nosema* and the revised genus name *Vairomorpha* are currently used in the literature [18,19,20,21,22,23,24]. Accordingly, in this study, both nomenclatures are used interchangeably for clarity and consistency with existing publications.

Within the Apidae family, *Nosema ceranae* was first identified as a pathogen in *Apis cerana* in 1996 [10], and was subsequently recognized as a novel pathogen of *Apis mellifera* [8,25]. Since then, both *N. apis* and *N. ceranae* have been worldwide, including in South American countries such as Brazil [26], Argentina [27], Chile [28], and Uruguay [29,30], as well as in the Dominican Republic [31], and northern North American countries such as Mexico [32], the USA, and Canada [33].

Infection occurs primarily through the ingestion of spores in contaminated food or during hive-cleaning activities [34,35,36,37]. *Nosema* infections are often chronic and may spread beyond the midgut, affecting multiple tissues and leading to subtle but progressive impairments in behavior, metabolism, and nutrition [38,39,40]. These alterations reduce worker longevity, increase colony mortality, and ultimately result in decreased production and colony losses, underscoring the importance of early and accurate detection of this pathogen [41,42].

Several diagnostic methods for *Nosema* infection have been described, including light microscopy, fluorescence microscopy, and molecular techniques [36,43]. The latter are the most commonly used because it is difficult to differentiate between the two *Nosema* species morphologically under a light microscope. PCR-based methods targeting the 16S rRNA gene are widely used for detection and phylogenetic analyses [14,44,45,46]. In addition, primers targeting the large subunit of the RNA polymerase II gene (RPB1) have proven effective for species differentiation, as well as for analyzing the population structure and genetic diversity of *Nosema* spp. [47,48,49,50]. Therefore, in this study, primers targeting the RPB1 gene were used for the molecular differentiation of *Nosema* species, while 16S rRNA gene sequences were employed for the phylogenetic analysis of *N. ceranae*.

Despite previous reports of *Nosema* in Ecuador, no molecular studies have confirmed the presence or identity of *N. ceranae*. This study aimed to detect, differentiate, and phylogenetically characterize *N. apis* and *N. ceranae* in honey bee colonies from the northern Ecuadorian highlands using multiplex PCR and sequence analysis.

## 2. Materials and Methods

### 2.1. Sample Collection

Based on the data obtained from the first beekeeping census carried out by AGROCALIDAD (2016) [1], the study area focused on the provinces of Pichincha and Imbabura, given the greater concentration of apiaries (a) and hives (h) in the northern part of the Ecuadorian Sierra, and the province of Carchi, because it is the border province with Colombia.

Between the months of April and June 2017, selected honey bee samples were collected from the hive entrances (h = 164) located in apiaries (a = 29) in the three studied provinces (Table 1).

Although the study was not aimed at determining risk factors related to the introduction and maintenance of *Nosema* sp. in hives and apiaries, stratified random sampling was used. Apiaries were selected based on a database of registered beekeepers provided by the AGROCALIDAD in the provinces included in the study. Beekeepers were contacted to assess their willingness to participate, and participating apiaries were further categorized according to the number of colonies managed. Within each selected apiary, a proportional number of colonies was sampled according to their developmental stage, including nucleus colonies, single-brood-chamber hives, double-brood-chamber hives, and double-chamber hives consisting of one brood chamber and one honey production chamber.

Inclusion criteria for apiary selection were as follows: (i) location within the study area, (ii) official registration in the AGROCALIDAD database, and (iii) informed consent to participate in the study. Exclusion criteria included the following: (i) multiple apiaries belonging to the same beekeeper within the same province, and (ii) beekeepers who did not complete the associated epidemiological survey.

### 2.2. Diagnostic Tests

For the diagnosis of *Nosema* sp. in honey bees, light microscopy and PCR laboratory tests were used. Each of the 164 samples was individually analyzed with both techniques. In addition, the fluorescence microscopy test was used on one of the samples diagnosed as co-infected by PCR to observe and compare the size of the *N. apis* and *N. ceranae* spores.

### 2.3. Optical Microscopy Test and Determination of Spore Number

The abdomens from approximately 20 honey bees per colony were aseptically separated with forceps and a scalpel, mixed with 1 mL of distilled water, macerated, and placed in vials. An aliquot of the sample (10 µL) was placed on a Neubauer chamber and visualized with an optical microscope at 400× magnification.

The spore concentration was obtained by multiplying the average number of spores in the sample by the dilution factor and dividing by the product of the chamber area (mm) by the chamber depth (mm). The level of bee infestation was then classified according to the following scale: low (<1,000,000 spores/bee), medium (>1,000,000 <2,000,000 spores/bee), and high (more than 2,000,000) [51].

### 2.4. DNA Extraction of Nosema *sp.* in Honey Bees

The protocol used for DNA extraction was as described by Hamiduzzaman et al. (2010) [52], with modifications. The abdomens of 20 honey bees from each colony were placed in 2 mL vials. A total of 500 µL of extraction buffer (0.03 M CTAB (PhytoTechnology Laboratories, Lenexa, KS, USA), 0.05 M Tris (Invitrogen, Carlsbad, CA, USA), 0.01 M EDTA (Invitrogen, Carlsbad, CA, USA), 1.1 M NaCl (Loba Chemie, Mumbai, India), pH 8.0) and 4 µL of Proteinase K (20 mg/mL, Invitrogen, Carlsbad, CA, USA) were added. Samples were triturated with a sterile pistil, vortexed, and incubated at 60 °C for 3 h with constant shaking, occasionally inverting the tubes during incubation. They were then centrifuged for 1 min at 16,000× *g*, and the supernatant was transferred to a 1.5 mL vial. A double extraction with phenol-chloroform (1:1) was performed by adding 300 µL of this mixture, homogenizing the tubes by inversion, and centrifuging them at 16,000× *g* for 15 min; the supernatant was transferred to a new vial. Then, 300 µL of chloroform (Merck, Darmstadt, Germany) was added and centrifuged at 8000× *g* for 5 min. 30 µL of 3 M sodium acetate (Loba Chemie, Mumbai, India) and 600 µL of 95% ethanol (Merck, Darmstadt, Germany) were added to the supernatant, mixed gently, and stored at −20 °C overnight. The samples were centrifuged at 8000× *g* for 10 min, and the ethanol was discarded. Subsequently, 1 mL of 75% ethanol (4 °C) was added and mixed briefly by vortexing. The pellet was then centrifuged for 3 min at 16,000× *g*, the ethanol was discarded, and the pellet was allowed to dry. Finally, the DNA pellet was re-suspended in 100 µL of UltraPure^TM^ DNase/RNase-Free Distilled Water (Invitrogen, Carlsbad, CA, USA), and the samples were incubated in a water bath at 65 °C for 10 min. Samples were incubated with RNAse (Invitrogen, Carlsbad, CA, USA) at 37 °C for 10 min. The extracted DNA was stored at −20 °C until use.

### 2.5. Detection and Identification of Nosema *sp.* by Multiplex PCR

DNA samples were analyzed by multiplex PCR, using two pairs of species-specific primers targeting different regions of the RPB1 gene (Table 2). The primers pair NosaRNAPol-F2/NosaRNAPol-R2 amplified a diagnostic fragment of approximately 297 bp for the detection of *N. apis*, whereas the primer pair NoscRNAPol-F2/NoscRNAPol-R2 generated an amplicon of approximately 662 bp for *N. ceranae*.

The multiplex PCR assay was optimized by adding 1× of PCR buffer, 0.5 µM of primers NosaRNAPol-F2/NosaRNAPol-R2 for the detection of *N. apis*, 0.4 µM of primers NoscRNAPol-F2/NoscRNAPol-R2 for *N. ceranae*, 1.75 mM MgCl2, 0.8 mM dNTP mix (0.2 mM/dNTP, Promega, Madison, WI, USA), 1.25 U/µL Taq polymerase enzyme (Invitrogen, Carlsbad, CA, USA), 400 ng DNA, and a volume of UltraPure^TM^ DNase/RNase-Free Distilled Water (Invitrogen, Carlsbad, CA, USA) to complete 25 µL of reaction. Cycling conditions in the thermal cycler ProFlex™ (Applied Biosystems, Foster City, CA, USA) were 95 °C initial denaturation for 5 min, 40 one-minute cycles of denaturation steps at 94 °C, annealing primer 67 °C, extension at 72 °C, and a final extension cycle at 72 °C for 10 min.

Positive controls (samples positive for *N. apis* and *N. ceranae*) and a negative control (water) were used in all reactions.

Additionally, a single PCR assay with 218MITOC-FOR and 218MITOC-REV primers (Table 2) was performed on *N. ceranae*-positive samples according to the results of the multiplex PCR. A 218–219 bp fragment of the 16S rRNA gene was amplified, following the protocol described by Higes et al. (2006) [8].

### 2.6. Sequencing, Molecular Characterization, and Phylogenetic Analysis

After molecular detection of *Nosema* species by multiplex PCR, phylogenetic analysis of *N. ceranae* was performed, as the species of greatest interest, based on the 16S rRNA gene primers (Table 2). Only those *N. ceranae*-positive samples with a strong band intensity were chosen, and the products of the single PCR assay were sent for sequencing, in duplicate and in both directions by the Sanger method to Macrogen^®^ (Seoul, South Korea). Consensus sequences (*n* = 9) from Ecuador were compared with sequences of isolates available in GenBank.

A phylogenetic tree was constructed to determine the phylogenetic relationship between *N. ceranae* isolates from Ecuador and sequences belonging to the Americas, Europe, and Asia, as well as to observe the relationship between the sequences from this study and other sequences within the *Nosema* genus. The tree was constructed using ClustalW algorithm as implemented in MEGA 12, with 1000 bootstrap replicates, based on the consensus sequence from this study and the sequences available in GenBank. The maximum parsimony (MP) method, which uses the subtree-pruning-regrafting (SPR) algorithm, was employed for the analysis. This analysis involved 24 nucleotide sequences. There was a total of 222 positions in the final dataset. *Trachipleistophora hominis* was used as the outgroup. Maximum parsimony is particularly suitable for first-time reports and species-level identification because the model does not require the specification of an a priori substitution model and instead groups sequences based solely on the minimal number of character changes [53].

### 2.7. Detection of Spores by Fluorescence Microscopy

Sample A27C2 was subjected to complementary analysis by fluorescence microscopy following a modified version of the protocol described by Snow (2016) [54]. Smear preparation of bee macerates was fixed by incubation with 60 µL of 3% glutaraldehyde (Sigma-Aldrich, St. Louis, MO, USA) for 2 h at room temperature. The fixative was removed by two washes of 10 min each with 1 mL of PBS-T solution (PBS (Invitrogen, Carlsbad, CA, USA) containing 0.01% Triton X-100 (Invitrogen, Carlsbad, CA, USA)). Samples were then stained with 500 μL of Calcofluor White stain (Fluorescent Brightener 28; Sigma-Aldrich, St. Louis, MO, USA) and incubated overnight at 4 °C in a humid chamber. After two additional washes with PBS-T, samples were counterstained with 200 μL of Hoesch DNA dye (1:2000 dilution; Invitrogen, Carlsbad, CA, USA) for 5 min at 4 °C in a humid chamber, followed by two final washes with PBS-T.

Slides were air-dried at room temperature in the dark and examined using an Olympus IX53 fluorescence microscope (Olympus, Tokyo, Japan) equipped with a 40× oil-immersion objective (NA 1.3). Fluorescence signals were detected using excitation wavelengths of approximately 365–405 nm for Calcofluor White and 350–365 nm for Hoechst, with a consistent exposure time of 384.6 ms for image acquisition.

## 3. Results

### 3.1. Detection of Nosema *sp.* Spores by Optical Microscopy

**Figure 1:**
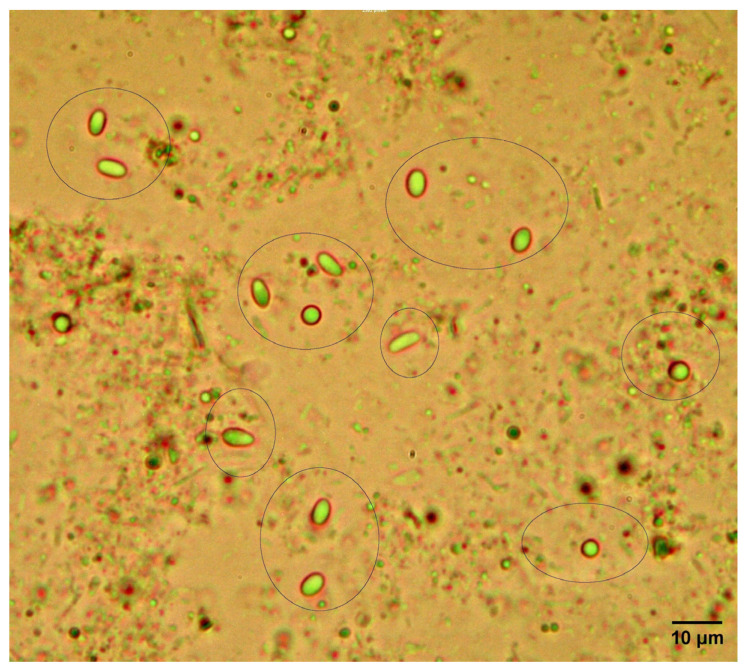
Spores (black circles) of *Nosema* sp. by optical microscopic examination (40×).

Microscopy revealed characteristic oval spores consistent with *Nosema* sp. morphology (Figure 1). The prevalence of *Nosema* spp. was 41.38% (12/29) at the apiary level and 17.07% (28/164) at the colony level.

The province of Pichincha (h = 63) had the highest number of positive samples (20/63), followed by the province of Carchi (5/33), and finally Imbabura (3/68) (Table 3). On the other hand, low (h = 9), medium (h = 2), and high (h = 17) levels of infestation or spore intensity were observed.

### 3.2. Identification of Nosema Apis and Nosema Ceranae by PCR

By multiplex PCR, we detected *Nosema* sp. infection in 34.76% (59/164) of colonies and 86.21% (25/29) of apiaries. We determined the presence of *N. apis* and *N. ceranae* in the colonies of the three provinces with a prevalence of 14.63% (24/164) and 21.34% (35/164, respectively, finding also apiaries (a = 5) and colonies (h = 2) with double infections.

Among the three provinces, Pichincha showed the highest prevalence of both *N. apis* and *N. ceranae* at the colony level (Table 3). Specifically, *N. ceranae* was identified in 36.51% of the colonies sampled in this province, exceeding the prevalence observed in Imbabura and Carchi.

**Figure A1:**
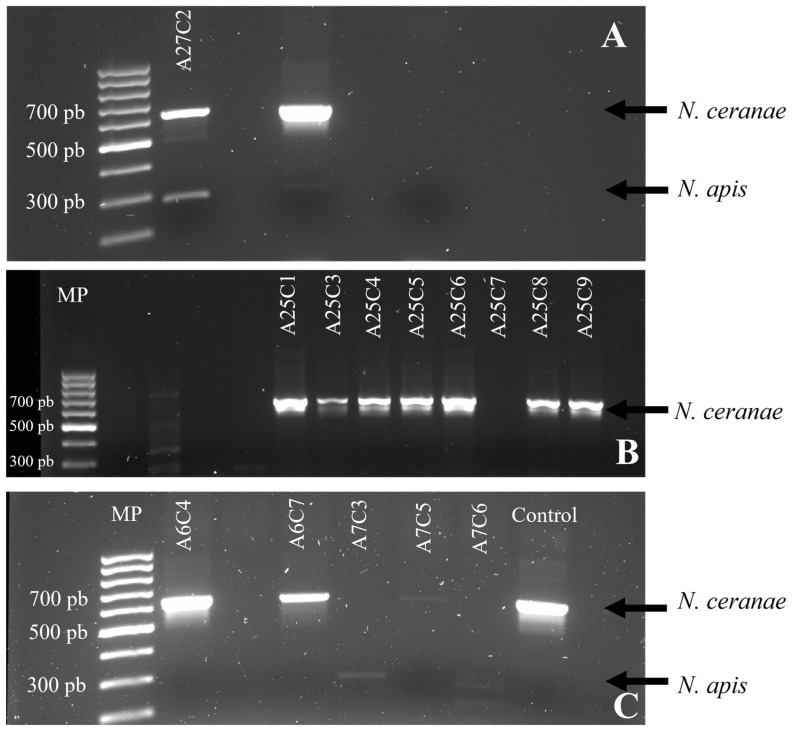
Detection results of *Nosema* sp. by multiplex PCR. Legend: (**A**) Sample A27C2 from Pichincha, hive with double infection by *N. apis* (300 pb approx.) and *N. ceranae* (between 600 and 700 pb). (**B**) Samples from Pichincha apiary with 7 hives (A25C1, A25C3, A25C4, A25C6, A25C8, A25C9) that were positive for *N. ceranae*. (**C**) Samples from two apiaries, apiary 6 with hives infected by *N. apis* and apiary 7 with hives infected by *N. apis* and *N. ceranae*.

Table 3 and Table 4 give details of the distribution of results (number, prevalence, and 95% confidence intervals) for light microscopy and PCR tests, at the apiary, colony, and province level. The PCR multiplex gel electrophoresis diagram is shown in the Appendix A (Figure A1).

### 3.3. Molecular Characterization and Phylogenetic Analysis of N. Ceranae

BLAST (Basic Local Alignment Search Tool, https://blast.ncbi.nlm.nih.gov/Blast.cgi?PROGRAM=blastn&PAGE_TYPE=BlastSearch&LINK_LOC=blasthome, accessed on 16 August 2025) analysis of the fragment sequence was 99.5–100% identity with partial sequences of small subunit ribosomal RNA gene isolates. Nine sequences of *N. ceranae* (*n* = 9) were obtained from various sectors of the provinces of Imbabura (*n* = 3) and Pichincha (*n* = 6), accession numbers PQ336918, PQ336919, PQ336920, PQ336921, PQ336922, PQ336923, PQ336924, PQ336925, PQ336926. Since all of them showed 100% homology, only one sequence was used in the phylogenetic tree (PQ336918).

**Figure 2:**
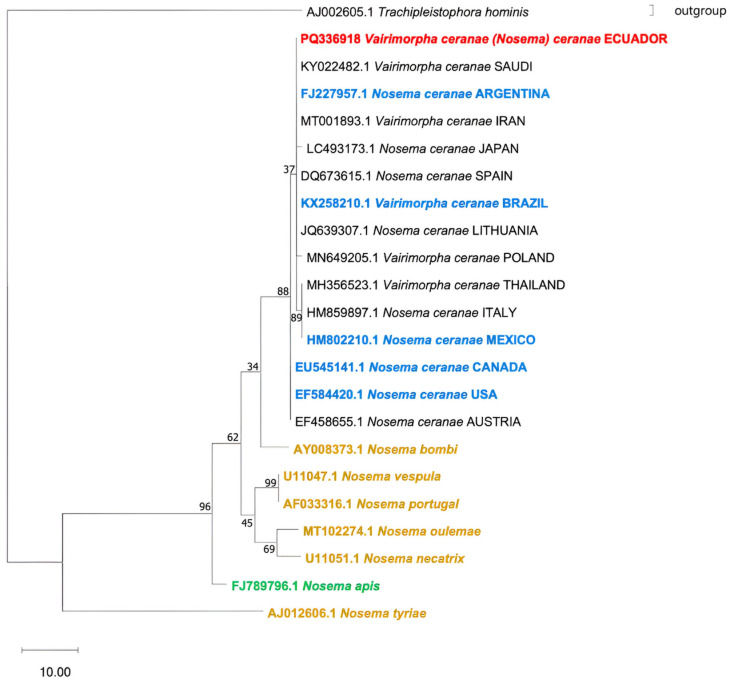
Phylogenetic tree of microsporidia *Nosema* based on the sequences of the small subunit rRNA and constructed by Maximum Parsimony analysis. Blue: *N. ceranae* from Americas; gold: other *Nosema* species; green: *N. apis*; black: *N. ceranae* from continents other than the Americas; red: *N. ceranae* from Ecuador. The percentage of replicate trees in which the associated taxa clustered together in the bootstrap test (1000 replicates) is shown next to the branches. The consistency index is (0.838), the retention index is (0.883), and the composite index is 0.783 (0.740) for all sites and parsimony-informative sites (in parentheses).

The phylogenetic analysis involved nine species of microsporidia belonging to this genus (Figure 2). We observed that the isolates from Ecuador are located within the same clade of *N. ceranae*, confirming that they belong to this species. Furthermore, the closest species are *N. bombi* and other *Nosema* species, leaving *N. apis* distantly related to *N. ceranae*.

Within the *N. ceranae* clade (Figure 2), we did not observe a grouped regionalization by continent. Thus, sequences from the American continent are distributed among all *N. ceranae* subclades. Consequently, the isolate from Ecuador is placed at the same phylogenetic distance as isolates from Argentina and Brazil (South America), Saudi Arabia and Iran (Asia), and Spain and Lithuania (Europe).

### 3.4. Detection of Nosema *sp.* by Fluorescence Microscopy

**Figure 3:**
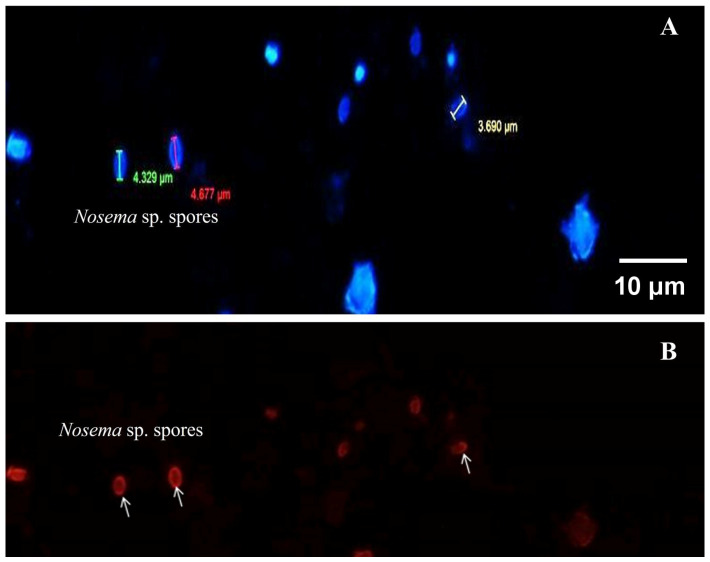
Fluorescence microscopy of *Nosema* spores detected in sample A27C2. Spores were stained with Calcofluor White (Fluorescent Brightener 28, blue color observed) and counterstained with Hoechst DNA dye (red color observed). Observations were performed using an Olympus IX53 fluorescence microscope with a 40× oil-immersion objective (Tokyo, Japan). Calcofluor White highlights the chitin-rich spore wall, while Hoechst staining confirms the presence of nucleic material.

Through fluorescence microscopy (Figure 3), the oval forms of *Nosema* sp. spores were visualized, measuring 4–6 μm in length, stained with Hoesch dye, which can stain the DNA of cells, and additionally, it was possible to take approximate measurements of them. The red staining of the spores indicates a positive result from the FB28 dye or calcofluor, which is specific for chitin, a polysaccharide component of the fungal cell wall. This specific staining allows us to confirm definitively that they are *Nosema* sp. microsporidia.

## 4. Discussion

This study is the first to apply molecular techniques for the diagnosis of pathogens in Ecuadorian apiaries. Molecular analyses confirmed the presence of *Nosema apis* and *Nosema ceranae*, and allowed their respective prevalence to be determined. These values are higher than those reported by AGROCALIDAD (9% of apiaries nationwide), which were based exclusively on microscopic observation and did not allow species-level differentiations [1].

The prevalence of *N. ceranae* determined in this study is consistent with reports from neighboring countries, such as Brazil, Argentina, and Chile, where this species has largely displaced *N. apis* or exhibits higher prevalence levels [26,27,28,29,30,40,44,55,56,57]. This study also identified apiaries and individual colonies with co-infections by *N. apis* and *N. ceranae*, as detected by multiplex PCR. Co-infections were observed at a lower prevalence than single infections, similar mixed infections have been reported in Turkey and Argentina [14,58]. Co-infections are epidemiologically relevant because they may influence parasite competition, infection dynamics, and host physiological response, potentially exacerbating colony-level impact [59,60]. The detection of coinfections in apiaries from Ecuador, therefore, highlights the need for diagnostic approaches capable of identifying mixed infections. Neither light microscopy nor fluorescence microscopy using calcofluor white can distinguish between *Nosema* species. The former is relatively straightforward and useful for preliminary screening [60], the latter uses calcofluor, which binds specifically to the chitin in the walls of mature spores, regardless of the species identity [54]. In contrast, multiplex PCR targeting the RPB1 gene demonstrated high sensitivity and specificity, enabling reliable discrimination between *N. apis* and *N. ceranae*. These results support previous findings emphasizing the superiority of molecular methods over other techniques for epidemiological surveillance [43,61,62] and underscore the need to incorporate PCR-based diagnostics into national regulatory and monitoring programs to improve accuracy in prevalence estimates and disease management strategies.

Phylogenetic analysis based on the 16S rRNA gene revealed that *N. ceranae* isolates from Ecuador are identical to sequences from the South American continent (Argentina and Brazil) as well as with isolates reported from Asia (Iran and Saudi Arabia) and Europe (Spain and Lithuania). Rather than indicating geographic structuring, this pattern could be consistent with a recent global expansion of *N. ceranae*, facilitated by the international trade in bees and bee products. Similar findings of shared or identical haplotypes in distant regions have been previously reported from samples originating in Spain, Slovenia, and Kyrgyzstan [63].

Likewise, molecular phylogenetic analyses indicate that *N. apis* and *N. ceranae*, despite infecting the same host species, are highly divergent and not closely related within the genus *Nosema*. This marked genetic separation supports the view that these microsporidia represent distinct evolutionary lineages with potentially different infection strategies, pathogenicity, and epidemiological dynamics [35,40,49].

While the scope of this study was necessarily focused on a limited geographic area, number of apiaries, and sampling period, it establishes an essential baseline for understanding the molecular epidemiology of *Nosema apis* and *Nosema ceranae* in Ecuador. The data generated here provide the first reference point for future investigations and contribute critical initial evidence to a field where information is currently scarce.

Building on this foundation, future epidemiological studies could expand coverage to Ecuador’s three natural regions to evaluate prevalence patterns, associated risk factors, and seasonal and interannual variability. In addition, longitudinal studies integrating socio-economic, productive, ecological, and case–control approaches would further clarify the significance and impact of *Nosema* spp. on honey bee and meliponine (native bee) apiaries in Ecuador.

## 5. Conclusions

This study is the first to report the presence of *Nosema ceranae* and *N. apis* in honey bee colonies in Ecuador. *N. ceranae* is more prevalent than *N. apis*, with co-infections detected at the colony level. The detection of co-infections highlights the potential for pathogen exchange within apiaries.

Phylogenetic analysis based on 16S rRNA sequences shows that *N. ceranae* isolates from Ecuador are identical to other isolates worldwide. This suggests that the commercialization of specimens and their products contributes to this phenomenon. These findings emphasize the importance of ongoing molecular surveillance and epidemiological mapping to develop effective control strategies in Ecuador.

Furthermore, future research should broaden its geographic scope, examine seasonal variations, and evaluate the impact of *Nosema* infections on colony health and productivity, in order to inform evidence-based management strategies.

## Figures and Tables

**Table 1:** Distribution of existing and sampled apiaries and beehives in the three provinces surveyed.

Province	Number and Percentage of Apiaries of the National Total ^a^	Number and Percentage of Apiaries Sampling	Number and Percentage of Beehives of the National Total ^b^	Number and Percentage of Beehives Sampling
**Carchi**	40 (4.43%)	4/40 (10%)	974 (7.99%)	33/974 (3.39%)
**Imbabura**	74 (8.20%)	13/74 (17.58%)	1025 (8.41%)	68/1025 (6.64%)
**Pichincha**	108 (11.97%)	13/108 (12.04)	2778 (22.79%)	63/2778 (2.28%)
**Total**	222/902 ^a^ (24.61%)	30/222 (13.51%)	4777/12,188 (39.19%) ^b^	164/4777 (3.43%)

^a^ Apiaries at national level; ^b^ Number of hives nationwide.

**Table 2:** Primers used for the detection and phylogenetic analysis of *N. apis* and *N. ceranae*.

Primer Name	Sequence (5′-3′)	Species	Fragment Size
NosaRNAPol-F2 *	AGCAAGAGACGTTTCTGGTACCTCA	*Nosema* *apis*	297 bp
NosaRNAPol-R2	CCTTCACGACCACCCATGGCA		
NoscRNAPol-F2 *	TGGGTTCCCTAAACCTGGTGGTTT	*Nosema* *ceranae*	662 bp
NoscRNAPol-R2	TCACATGACCTGGTGCTCCTTCT		
218MITOC-FOR **	CGGCGACGATGTGATATGAAAATATTAA	*Nosema* *ceranae*	218–219 bp
218MITOC-REV	CCCGGTCATTCTCAAACAAAAAACCG		

bp: base pairs; * Primers for amplification of RPB1 gene fragments [47]; ** Primers for 16S rRNA gene fragment amplification [8].

**Table 3:** Detection of *Nosema* spp. in hives by microscopy and multiplex PCR.

Province	Total Hives	Microscopy	PCR		
		*Nosema* spp. Prevalence % (95% CI)	*N. apis* Prevalence % (95% CI)	*N. ceranae* Prevalence % (95% CI)	Co-Infection Prevalence % (95% CI)
**Carchi**	33	6 (18.18%) (6.98–35.46)	6 (18.18%) (6.98–35.46)	3 (9.09%) (1.92–24.33)	1 (3.03%) (0.08–15.76)
**Imbabura**	68	3 (4.41%) (0.92–12.36)	10 (14.71%) (7.282–25.39)	9 (13.24%) (6.33–23.64)	0 - *
**Pichincha**	63	19 (30.16%) (19.23–53.02)	8 (12.70%) (5.65–23.50)	23 (36.51%) (24.73–49.6)	1 (1.59%) (0.04–8.53)
**Total**	164	28 (17.07%) (11.65–23.72)	24 (14.63%) (9.61–20.99)	35 (21.34%) (15.34–28.41)	2 (1.22%) (0.15 4.34)

Note: Values are expressed as number of positive samples followed by prevalence (%) and 95% confidence intervals (CI). *: CI does not apply.

**Table 4:** Detection of *Nosema* spp. in apiaries by microscopy and multiplex PCR.

Province	Total Apiaries	Microscopy	PCR		
		*Nosema* spp. Prevalence % (95% CI)	*N. apis* Prevalence % (95% CI)	*N. ceranae* Prevalence % (95% CI)	Co-Infection Prevalence % (95% CI)
**Carchi**	4	2 (50%) (6.76–93.24)	3 (75%) (19.41–99.37)	1 (25%) (0.63–80.59)	1 (25%) (0.63–80.59)
**Imbabura**	13	3 (23.08%) (5.04–53.81)	5 (38.46%) (13.86–68.42)	7 (53.84%) (19.22–74.87)	0- *
**Pichincha**	12	7 (58.33%) (27.67–84.83)	4 (33.33%) (9.92–65.11)	8 (66.67%) (34.89–90.08)	2 (8.33%) (0.21–38.48)
**Total**	29	12 (41.38%) (23.52–61.06)	12 (41.38%) (25.52–61.06)	15 (51.72%) (32.53–70.55)	2 (6.90%) (0.85–22.77)

Note: Values are expressed as number of positive samples followed by prevalence (%) and 95% confidence intervals (CI). *: CI does not apply.

## Data Availability

The link and DOI for the database are as follows: https://data.mendeley.com/datasets/g7zt7g73fk/1, https://doi.org/10.17632/g7zt7g73fk.1. Accessed 18 December 2025.

## Abbreviations

GISAH	Grupo de Investigación en Sanidad Animal y Humana
RPB1	RNA Polymerase II Subunit RPB1
16S rRNA	16S ribosomal RNA
PCR	Polymerase Chain Reaction
*N. apis*	*Nosema apis*
AGROCALIDAD	Agencia de Regulación y Control Fito y Zoosanitario (Ecuador)
*N. ceranae*	*Nosema ceranae*
OIE	World Organization for Animal Health
GPS	Global Positioning System
DNA	Deoxyribonucleic Acid
EDTA	Ethylenediaminetetraacetic Acid
RNAse	Ribonuclease
dNTP	Deoxyribonucleoside Triphosphate
RNA	Ribonucleic Acid
USA	United States of America
